# Ocular manifestations of emerging viral diseases

**DOI:** 10.1038/s41433-020-01376-y

**Published:** 2021-01-29

**Authors:** Ashwin Venkatesh, Ravi Patel, Simran Goyal, Timothy Rajaratnam, Anant Sharma, Parwez Hossain

**Affiliations:** 1 School of Clinical Medicine, University of Cambridge, Cambridge, UK; 2 Moorfields Eye Hospital, London, UK; 3 Eye Unit, University Hospitals Southampton NHS Foundation Trust, Southampton, UK; 4 Clinical Experimental Sciences, Faculty of Medicine, Univeristy of Southampton, Southampton, UK

**Keywords:** Eye manifestations, Microbiology

## Abstract

Emerging infectious diseases (EIDs) are an increasing threat to public health on a global scale. In recent times, the most prominent outbreaks have constituted RNA viruses, spreading via droplets (COVID-19 and Influenza A H1N1), directly between humans (Ebola and Marburg), via arthropod vectors (Dengue, Zika, West Nile, Chikungunya, Crimean Congo) and zoonotically (Lassa fever, Nipah, Rift Valley fever, Hantaviruses). However, specific approved antiviral therapies and vaccine availability are scarce, and public health measures remain critical. Patients can present with a spectrum of ocular manifestations. Emerging infectious diseases should therefore be considered in the differential diagnosis of ocular inflammatory conditions in patients inhabiting or returning from endemic territories, and more general vigilance is advisable in the context of a global pandemic. Eye specialists are in a position to facilitate swift diagnosis, improve clinical outcomes, and contribute to wider public health efforts during outbreaks. This article reviews those emerging viral diseases associated with reports of ocular manifestations and summarizes details pertinent to practicing eye specialists.

## Abstract (zh)

新兴传染性疾病 (EIDs) 对全球公共卫生领域造成越来越大的威胁。近期, 由RNA病毒导致的爆发性传染病尤为突出, 其可以通过飞沫传播 (COVID-19和甲型H1N1流感) 、人与人直接接触传播 (埃博拉和马尔堡) 、虫媒传播 (登革热、兹卡、西尼罗河、基孔肯亚、克里米亚-刚果) 和动物传播 (拉沙热、尼帕、裂谷热、汉坦病毒) 。然而, 特批的抗病毒治疗和疫苗稀缺, 所以公共卫生工作仍至关重要。患者可出现一系列眼部的表现。因此, 若患者从流行地区返回或居住在流行地区时, 该患者眼部炎症情况的鉴别诊断应考虑到新兴传染病, 尤其在全球大流行的情况下, 更应普遍地提高警惕。眼科专家的快速诊断, 对临床预后的改善, 在疫情爆发期间对公共卫生工作做出更广泛的贡献。本文回顾了与眼部症状相关的新兴病毒性传染病的临床表现, 并总结了与眼科专家有关的诊治细节。

## Introduction

Emerging infectious diseases (EIDs) pose a considerable ongoing threat to humanity. The World Health Organization (WHO) define EIDs as those that are either novel and severely affect a population for the first time, or those that have existed previously but have a rapidly increasing incidence or degree of spread to new geographical areas [1]. Globalisation and climate change have led to an acceleration in the emergence of EID outbreaks in recent times, with the WHO reporting 1483 epidemic events in 172 countries between 2011 and 2018 [2]. Since infectious diseases are increasingly extending beyond endemic territories, the clinician should be armed with a global perspective.

Recent outbreaks combined with the development of diagnostic techniques have increasingly allowed characterisation of the ocular manifestations of EIDs [3]. The most prominent outbreaks in recent times have constituted viruses such as the 2014–2016 Ebola outbreak in West Africa, 2015–2016 Zika outbreak in the Americas, a series of increasingly frequent Dengue outbreaks and the current severe acute respiratory syndrome coronavirus 2 (SARS-CoV-2) pandemic [4–7]. With the necessary precautions, eye specialists are therefore in a position to facilitate swift diagnosis, improve clinical outcomes and contribute to wider public health efforts during outbreaks. To this end, we provide a timely review of emerging viral diseases for the ophthalmologist (Table 1).

**Table 1:** Summary table of emerging viral diseases with ocular manifestations, detailing their epidemiology and presentation, and grouped according to primary mode of transmission. Emerging viral diseases with ocular manifestations.





Emerging viral diseases with ocular manifestations.

## Methodology

Emerging viral diseases were identified for inclusion using the WHO list of designated ‘priority’ pathogens as well as an initial scoping literature search for emerging viral diseases with ocular manifestations [8]. For each virus identified for inclusion, an in-depth literature search was performed using MEDLINE’s database, with variations on virus name, emerging disease, and ocular manifestations, as key search terms. Studies selected were in English and pertaining to humans. Searches were supplemented by reviewing reference lists of relevant papers. The viruses discussed will be grouped according to the mechanism of transmission and family.

## Human-to-human

### Ebola* (Filoviridae)

Ebola virus (EBOV) is an enveloped, filamentous, negative-sense, single-stranded RNA virus of the Filoviridae family. EBOV is the cause of the frequently lethal Ebola virus disease (EVD), which has a documented case fatality rate of around 50% [9]. EVD outbreaks typically start from a single case of probable zoonotic transmission, and fruit bats, likely of the Pteropodidae family, are believed to be the natural reservoir for the virus. Human-to-human transmission then occurs via direct contact, or contact with infected bodily fluids or contaminated fomites. The unprecedented 2013–2016 Western African EVD outbreak (the largest in history: 28,600 cases and 11,300 deaths) and the ongoing EVD outbreak in the Democratic Republic of the Congo have shed light on the spectrum of manifestations of EVD [10].

After contact with the virus, the asymptomatic incubation period ranges from 2 to 21 days with an average of 8 to 10 days. EVD is typically characterized by an initial fever, myalgias and fatigue, progressing to gastro-intestinal symptoms (diarrhoea and vomiting) and eventually a fatal multiple organ dysfunction syndrome. During acute infection, ocular manifestations include conjunctival injection (48–58%; often bilateral), subconjunctival haemorrhages and vision loss of unclear origin [4].

**Fig. 1:**
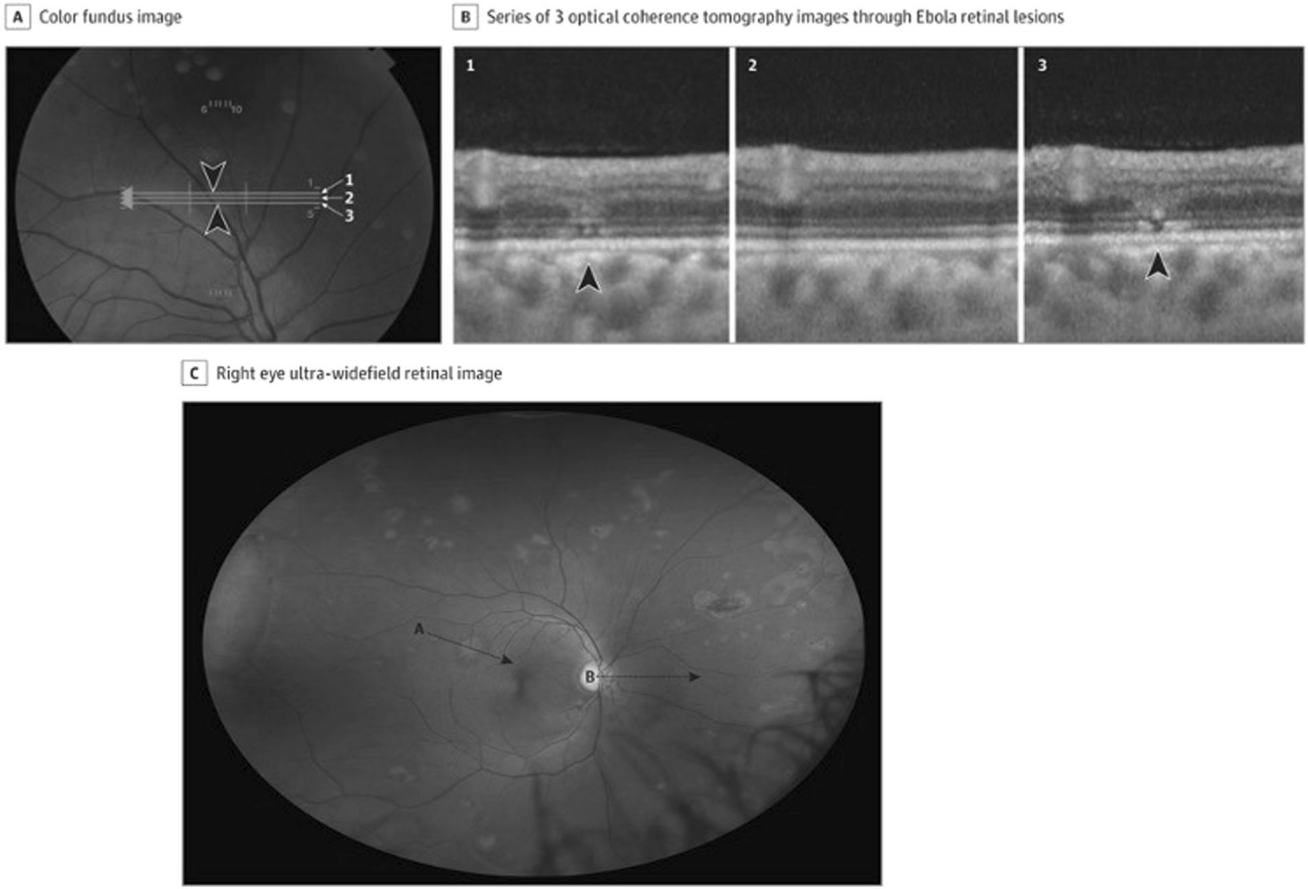
Ebola retinal lesions. **A** Colour fundus image, **B** corresponding OCT images showing discontinuities in the outer retinal layers, **C** multiple non-pigmented lesions and associated perilesional areas of dark without pressure. Source: Steptoe et al. [18] (CC-BY License).

An important clinical sequel has surfaced in survivors, termed the ‘post-Ebola virus disease syndrome’. This commonly manifests with ocular disease (60%), and may be accompanied by arthritis, hearing loss, abdominal pain and neuropsychiatric disorders as well as viral persistence in immune-privileged organs [11]. Uveitis is the most common ocular complication, occurring in ~13–34% of EVD survivors in West Africa within the first 12 weeks (but sometimes even after a year) following convalescence [12]. This commonly presents with eye pain, redness and photophobia and may lead to acute or chronic vision loss, with a unilateral predominance [13]. Within uveitis, case series quote variable prevalence statistics, overall indicating anterior uveitis as most frequent, followed by posterior uveitis, and rarely panuveitis [14–16]. Slit lamp examination often shows non-specific signs of active or past ocular inflammation e.g. posterior synechiae, keratic precipitates, anterior chamber cell and flare and conjunctival injection [17]. Multimodal imaging including fundus photography and optical coherence tomography (OCT) can be used to evaluate retinal lesions in posterior or panuveitis. A case series evaluating 14 EVD survivors noted retinal lesions that were predominantly non-pigmented with perilesional areas of dark without pressure, and peripapillary lesions that exhibited variable curvatures respecting the horizontal raphe and sparing the fovea [18]. OCT showed abnormalities of the outer retinal layers (Fig. 1). In these uncontrolled cohorts, risk factors predicting uveitis include conjunctival injection, and a high viral load (corresponding to a low cycling threshold on reverse transcription PCR (RT-PCR) analysis) during acute EVD, and advanced age [11, 14]. The PREVAIL III prospective cohort study in Liberia found that survivors exhibited more frequent uveitis than controls, and the prevalence increased during follow-up (at enrolment, 26.4% vs. 12.1%; at year 1, 33.3% vs. 15.4%) [19]. Neuro-ophthalmic complications are also possible, including optic neuropathy and ocular motility disorders [16]. Other ophthalmic sequelae include episcleritis, interstitial keratitis and cataract [4, 14, 15].

Diagnosis requires a combination of case definition and laboratory tests, typically real-time RT-PCR or enzyme-linked immunosorbent assay (ELISA). This permits the swift initiation of appropriate cycloplegic and anti-inflammatory treatment (topical or systemic steroids depending on severity) for uveitis and recognition and management of complications. Notably, the finding of replicating EBOV in the aqueous humour following recovery from EVD (and for at least 100 days post acute EVD diagnosis) highlights possible infectivity of ocular fluid posing risk to ophthalmologists performing invasive procedures on EVD survivors [20, 21].

Timely diagnosis can help avoid long-term visual disability. Specifically, whilst USA healthcare workers swiftly treated recovered vision following therapy, nearly 40% of eyes in a Liberian cohort in whom treatment was comparatively delayed had vision worse than 20/400 than their initial presentation, sometimes because of structural complications including cataract and dense vitreous opacity [4]. In one study of 57 patients with uveitis after EVD, 7 (12%) were also diagnosed with cataracts concurrently with uveitis, and at least 3 others developed cataract(s) following the onset of uveitis [11]. This illustrates that interventions to counteract the resource-limited delays in uveitis diagnosis and treatment focussed in areas at-risk for EVD might help to reduce the risk of vision-related morbidity in future outbreaks. Recent evidence highlights the feasibility of establishing screening eye clinics for EVD survivors to facilitate this objective [22].

The mainstay of treatment for the underlying EBOV infection is currently supportive and symptomatic; however, antiviral drugs are emerging [23]. An experimental Ebola vaccine is now FDA-approved for the prevention of EVD in individuals aged 18 years and older [24]. It is being used in a ring vaccination protocol to control current outbreak in the Democratic Republic of the Congo [9].

### Marburg* (Filoviridae)

Marburg virus (MARV) is a negative-sense RNA virus of the Filoviridae family. Zoonotic infection occurs following prolonged exposure to *Rousettus aegyptiacus* bats, the natural reservoir of MARV, with outbreaks mostly endemic to East and Central Africa [25]. Secondary human-to-human transmission occurs primarily via direct contact with a symptomatic individual or their infected bodily fluids and MARV is known to persist in immune-privileged sites following recovery [26, 27]. After an incubation period of 2–21 days, Marburg virus disease (MVD) typically presents abruptly with high fever, severe headache and malaise, often with gastro-intestinal disturbance and a maculopapular non-pruritic rash. The development of haemorrhagic manifestations between 5 and 7 days signals late stage disease and case fatality rates between 23 and 90% have been recorded [28]. Ocular involvement is limited to a single case report of acute anterior uveitis developing during convalescence (3 months following acute MVD onset), which was successfully treated with topical steroids, atropine and acetazolamide [29, 30]. MARV can persist after resolution of symptoms or death in various human tissues, secretions and even immune-privileged sites. This is a finding supported more recently by primate studies highlighting viral persistence in the eye [27, 31]. Diagnosis is made by RT-PCR from blood or buccal swab, and a negative test does not rule out infection until symptoms have been present for at least 72 h [32]. There is no specific antiviral treatment or vaccine at present; aggressive supportive care and fluid resuscitation complemented by public health measures form the mainstay of management [27, 33].

### Human herpes virus-6 (HHV-6)

HHV-6 is a double-stranded DNA virus of the β-herpes virus subfamily found worldwide, with two variant species HHV-6A and HHV-6B. Little is known about the acquisition or prevalence of HHV-6A and it is less associated with disease [34]. In contrast, HHV-6B, which has a seroprevalence approaching 100%, with infection typically occurring in childhood [35]. The virus replicates in the salivary glands and is transmitted through the saliva. HHV-6 mRNA has been detected in the ocular fluid sample of a patient with uveitis suggesting that viral replication may occur in the eye [36]. Additional tests for HHV-6 RNA or protein in ocular tissues would provide more definitive evidence of this.

There are typically three stages in the natural history of HHV-6 infection [35]. Acute primary infection in childhood causes exanthem subitum [37], also called roseola infantum. This is characterised by a high fever followed by a rash that spontaneously resolves. Complications include CNS invasion, hepatitis and pneumonitis, in both immunocompetent and immunosuppressed patients. The second stage occurs in healthy adults and children where the virus remains latent in lymphocytes and monocytes and stays at a low level in tissues. The third stage is due to reactivation from latency or reinfection in immunosuppressed patients.

Ocular conditions of the posterior segment are more commonly associated with HHV-6, though the exact role of HHV-6 in these conditions remains unclear. Posterior segment inflammation associated with HHV-6 includes AIDS-associated retinitis [38–41], uveitis [36, 42–46] and optic neuritis [46–49]. The prevalence of HHV-6 in ocular fluid samples of patients with ocular inflammation has been found to be 2% or less [36, 42, 43]. The largest study to date found 7/350 patients with uveitis or endophthalmitis tested positive for HHV-6 DNA in ocular fluid samples [36]. The detection of HHV-6 DNA in the eye may not be clinically relevant as all seven patients were found to have other infectious agents in ocular fluid samples. Therefore, it is assumed that HHV-6 infections play a secondary role in the pathogenesis of ocular inflammation [36]. Presence of HHV-6 DNA can be accounted for as it latently resides in immune cells [35], which may enter the eye during inflammation due to destruction of the blood–retinal barrier [36]. Alternatively, HHV-6 DNA detected in intraocular fluids may be due to release of HHV-6 DNA from resident ocular cells caused by intraocular inflammation and this is supported by findings that HHV-6 can infect human retinal pigment epithelial cells [50].

In addition, reactivation of HHV-6 frequently accompanies CMV reactivation [51]. In nine patients with AIDS-associated retinitis, three were positive for HHV-6 and all three had simultaneous CMV coinfection [41]. This suggests that presence of HHV-6 may simply reflect the immunocompromised state of the patient.

Anterior segment disease associated with HHV-6 includes corneal inflammation [36, 52, 53]. HHV-6 was detected in 14/22 patients with corneal inflammation, specifically, dendritic keratitis, corneal ulcer or keratouveitis. This study showed the association of HHV-6 with disease is more frequent than other herpesviruses suggesting that HHV-6 may be another sole causative agent for corneal inflammation [52].

Diagnosis of clinically relevant HHV-6 is challenging due to the high prevalence of infection and need to distinguish between active and latent infections [36]. Detection of HHV-6 DNA in the plasma or serum indicates active infection [54]. Quantitative PCR can distinguish between active and latent infections of clinical samples tested, such as aqueous humour, vitreous or corneal samples. Multiplex PCR of the aqueous humour should be performed in suspected cases for early diagnosis of HHV-6 and specific antiviral therapy initiation [45, 46]. Foscarnet, ganciclovir, either alone or in combination and cidofovir should be used for management of HHV-6-related neurological disease and seems to be more efficient than acyclovir [45, 55, 56].

## Animal-to-human

### Lassa fever (LF)* (Arenaviridae)

Lassa virus (LASV) is an enveloped, negative-sense, single-stranded, bi-segmented RNA virus of the Arenaviridae family and is endemic to West Africa. Infection often occurs through exposure to food or household items contaminated with urine or faeces of infected Mastomys rats. Person-to-person transmission may occur after exposure to virus in bodily fluids, particularly in healthcare settings in the absence of control measures. Following a 7–21-day incubation period, LASV can cause Lassa fever (LF), an acute viral haemorrhagic illness with an overall case fatality rate of 1%, though most-affected individuals harbour the infection asymptomatically (80%) [57]. Ocular involvement is not extensively reported for LF, with a single case series noting conjunctivitis and conjunctival oedema in acute LF, whilst transient blindness has been documented during convalescence [58–60]. Animal studies highlight high viral loads present in the aqueous humour in rhesus macaques that died of experimental LASV infection [30]. LF is most often diagnosed by ELISA to detect IgM and IgG antibodies as well as Lassa antigen. Ribavirin seems to be an effective antiviral therapy early in the disease course, but its use as post-exposure prophylaxis is unsupported [61, 62]. There is currently no vaccine that protects against LF. Together, these findings support minimal long-term effects on vision in LF—further longitudinal studies are necessary to clarify this association, and in the meanwhile careful ophthalmic observation of LF survivors is recommended.

### Nipah* (Paramyxoviridae)

Nipah virus is an enveloped, negative-sense, single-stranded RNA virus in the family *Paramyxoviridae* of the genus *Henipavirus*. It was first isolated during the 1999 outbreak in Malaysia and Singapore. Repeated outbreaks have since occurred in India and Bangladesh. Transmission is primarily zoonotic from contact with infected pigs or bats, though person-to-person transmission has also been reported in Bangladesh and India, highlighting its pandemic potential [63]. There are currently no studies on viral persistence in bodily fluids.

The disease has a high mortality rate of 40% (Malaysia) to over 70% (India) [63]. Variation in mortality rates and clinical features in the two regions are likely due to different strains, which have separately co-evolved. The incubation period is from 4 to 21 days, followed by up to 2 weeks of fever and headache. Fatal encephalitis is the main complication of the disease and acute respiratory disease occurs to varying degrees.

Regarding ocular manifestations, one small study from Singapore of 13 patients reported nystagmus, VI nerve palsy and transient blindness during the acute phase of the illness [64]. Residual clinical signs at an 18-month follow-up included nystagmus, branch retinal artery occlusion, VI nerve palsy and Horner’s syndrome [64]. Retinal artery involvement is consistent with the hypothesis of vasculitic small-vessel infarction. Another study found that abnormal doll’s-eye reflex and pin-point pupils with variable reactivity were associated with a higher mortality suggesting greater brain stem involvement [65]. These two studies examined symptoms in pig farmers in Singapore or Malaysia. Therefore, further characterisation of ocular manifestations of the more pathogenic viral strains in Bangladesh and India is needed [66].

Diagnosis focuses on RT-PCR of throat swabs, blood, urine and CSF [67]. There is no approved vaccine and treatment is largely supportive with evidence for ribavirin treatment being equivocal [65, 68].

### Rift valley fever (RVF)* (Phenuiviridae)

RVF virus is an enveloped, negative-sense, single-stranded RNA virus, of the family Phenuviridae [69]. It is the cause of RVF, a zoonotic arthropod-borne disease, mainly affecting cattle. With an incubation period of 2–6 days, most human infections follow contact with the blood or organs of infected animals, typically occupationally. A smaller number occurs via mosquito bite (Aedes or Eretmapodites genus), and there are no documented cases of human-to-human transmission [70, 71]. Outbreaks, closely related to high-rainfall conditions, have mainly occurred in sub-Saharan Africa and North Africa, though more recently cases were reported in the Arabian Peninsula. In the five most recent outbreaks for which relevant data were recorded, an estimated total of 339,000 infections are believed to have occurred [72]. Documented case fatality rates are highly variable, though it is thought to be <1% [71].

In its most common mild form, RVF is characterised by fever and non-specific flu-like symptoms. In a small percentage of cases, a severe form develops, categorised into three syndromic presentations: ocular disease with risk of permanent loss of vision; meningoencephalitis with confusion and potentially coma; and haemorrhagic fever with liver involvement and jaundice, this being the most fatal form [73].

**Fig. 2:**
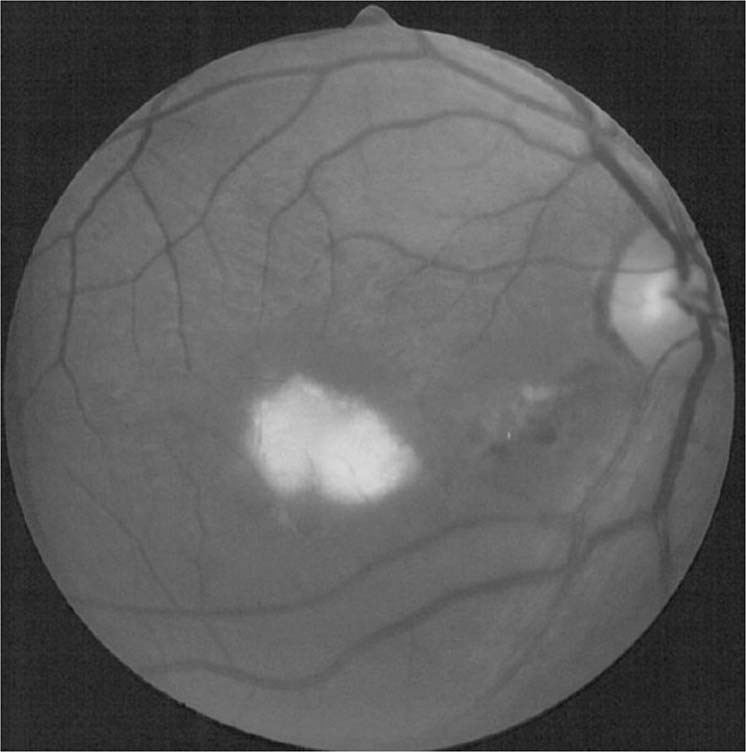
Rift Valley fever virus, fundus photograph, demonstrating active retinitis in the macular region and retinal haemorrhages. Source: Khairallah [231] (CC-BY licence), original publication Al-Hazmi 2005.

Ocular manifestations are well-documented, and present symptomatically with blurred vision, decreased vision, floaters, scotomatous areas or periocular pain [74–82]. The classical manifestation is of macular or paramacular retinitis (Fig. 2). It is difficult to estimate the true incidence of ocular complications in RVF, as total case numbers in outbreaks are not known and incidences in case series from different outbreaks vary significantly. However, the WHO estimates an incidence of 0.5–2% patients [71]. The largest series to date looked at 143 patients (212 eyes) all with macular or paramacular retinitis and serologically proved RVF in the 2000 Saudi Arabian outbreak [75]. Onset of visual symptoms ranged from 4 to 15 days. Associated lesions included retinal haemorrhages (40% of eyes), vitreous retractions (26%), optic disc oedema (15%) and retinal vasculitis (7%, mostly phlebitis, occasionally arteritis). A single creamy-white area of retinal necrosis was typically seen on fundoscopy reflecting retinitis, alongside the other described associated lesions. Fluorescence angiography demonstrated an early hypofluorescence of the area of retinitis and delayed filling of arterioles and venules, with late staining of the retinal lesion and blood vessels. Active lesions largely resolved over a 9-month follow-up, leaving behind chorioretinal scarring, and in some cases vascular occlusion and optic atrophy. Seventy-one percent of eyes remained legally blind at the end of follow-up, compared to 40–50% permanent visual loss reported in previous studies. Fifty-one eyes also developed a transient mild-to-moderate anterior uveitis, which appeared non-granulomatous.

RVF and its ocular manifestations may mimic other viral diseases, and so diagnosis relies on laboratory testing with either RT-PCR or serology [73]. No positive RVF viral cultures or RT-PCR have been reported as yet in CSF, or on aqueous or vitreous samples [83]. No disease-specific treatments are available, and no vaccines are licenced or commercially available for human use at present [71]. Severe cases are managed supportively.

### Hantaviruses e.g. Hantaan, Puumala, Seoul, Dobrava (Bunyaviridae)

Hantaviruses are enveloped, single-stranded, negative-sense RNA viruses of the Bunyaviridae family. The viruses are rodent-borne and transmission is via aerosols of excreta, saliva and urine, leading to an initially non-specific viral presentation of pyrexia, chills and myalgias following an incubation period (10–25 days). Person-to-person transmission is generally rare for hantaviruses, with the notable exception of Andes virus (ANDV) following reports of transmission of ANDV in the hantavirus cardiopulmonary syndrome (HCPS) outbreak in Argentina in 1996 [84, 85]. Nowadays, hantavirus infections are subdivided into two groups representing the two most common clinical patterns of infection [86–88]. ‘New World’ hantaviruses are principally found in the Americas, and may cause hantavirus cardiopulmonary syndrome (HCPS), characterized by interstitial pulmonary oedema leading to shock and cardiopulmonary failure. ‘Old World’ hantaviruses are found mostly in Europe and Asia, and may cause haemorrhagic fever with renal syndrome (HFRS), marked by sudden and extreme albuminuria due to renal medullary damage, that may lead to renal failure.

Transient ocular involvement in hantavirus infection is now increasingly recognised, and to date, has mainly been described in the context of HFRS caused by Puumala virus. An acute, transient myopia may be the first and most cardinal ocular symptom of a hantavirus infection, and its incidence has been variably reported (12–53%) in Puumala virus infections in Europe [89–91]. The underlying mechanism is unclear, but the leading theory involves an anterior shift of the lens due to underlying inflammation of the ciliary body [91]. Anterior shift of the lens may predispose to angle closure glaucoma, reported in exceptional cases, and may be associated with ciliochoroidal effusion [89, 92]. In the largest case series (46 patients) to date reporting on ocular involvement in patients who had recovered from acute HFRS, 70% reported ocular symptoms during the disease course, which were often bilateral [93]. During the acute phase of infection, 88% experienced decreased intraocular pressure, 87% reduced visual acuity, 87% conjunctival chemosis, 82% thickening of the lens, 78% myopic shift, 64% shallowing of the anterior chamber and 52% shallowing of vitreous length. Aside from anterior segment changes, macular features such as dot and blot intraretinal haemorrhages and streak haemorrhages of the disc have been reported on fundus imaging [94]. Recently, the first case report of presumed hantavirus necrotizing retinitis concurrent with HFRS was confirmed by fluorescein angiographic and funduscopic findings, and in this patient visual acuity losses improved months after resolution of the infection [95]. Together, these findings highlight that a diverse range of transient ocular disturbances in hantavirus infection are probable, and generally resolve with recovery from systemic infection.

At present, there are currently no approved treatments or vaccines available for hantavirus infections [96]. Supportive management in the intensive care unit is the current mainstay of treatment, with better patient outcomes associated with ICU admission earlier in the disease course (CDC).

## Arboviruses

### Dengue (Flaviviridae)

Dengue viruses are enveloped, positive-sense, single-stranded, RNA viruses of the Flaviviridae family. They are the cause of dengue fever. There are four viral serotypes 1–4 with no cross-immunity, and spread is via Aedes mosquito vectors [97]. Incidence has increased 30-fold over recent decades, and there are now an estimated 96 million symptomatic infections annually, two million cases of severe dengue, and 21,000 deaths [98–101]. Occurring in endemic–epidemic cycles in crowded tropical urban areas, the largest numbers of infections are in children in Asia and young adults in the American tropics, albeit with significant extension to other continents [102].

Dengue fever occurs 3–14 days after a mosquito bite, with development of a non-specific febrile phase lasting 3–6 days [97]. Upon fever resolution, some cases progress to a critical phase of plasma leakage into abdominal and pleural cavities, also sometimes associated with impaired haemostasis. Other findings include vomiting, petechial rash, myalgia, hepatomegaly, lymphopenia and thrombocytopenia. A convalescent phase follows. Cases of severe dengue involve dengue shock syndrome and respiratory distress secondary to plasma leakage, severe bleeding and severe end organ involvement [98].

True incidence of ocular manifestations is poorly understood, with studies only including cases that reach hospital specialists. In such studies incidence has been reported between 7.1 and 40.3%, a range that likely reflects differing disease severities and differing ocular workups in each study [103, 104]. Ocular manifestations occur from days to months after dengue fever onset. Reported anterior segment manifestations include, more commonly, subconjunctival haemorrhages and anterior uveitis, and more rarely, shallow anterior chambers, acute angle closure glaucoma, superficial punctate erosions, keratitis, vitritis, intermediate uveitis and scleritis [6, 104, 105]. Anterior uveitis has been reported to present with eye pain, redness, photophobia, ciliary injection, low-grade anterior chamber cells and diffuse keratic precipitates [81].

**Fig. 3:**
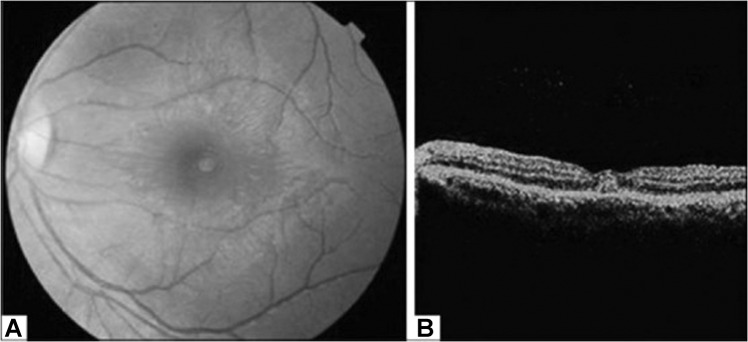
Dengue foveolitis in left eye. **A** Fundus photograph showing orange-yellow central foveal lesion **B** OCT scan demonstrating focal thickening of the outer neurosensory retina and retinal pigment epithelium. Source: Khairallah [231] (CC-BY licence), original courtesy of Soon-Phaik Chee.

Posterior segment manifestations most commonly include maculopathy and posterior uveitis, and less commonly vascular occlusions, panuveitis and retinal and vitreous haemorrhage [6]. Maculopathy, typically asymmetric and bilateral, is reported in 10% of hospitalised patients, and is serotype-dependent [106, 107]. Onset of symptoms in maculopathy, such as decreased visual acuity and a central scotoma, occur 3–11 days after fever onset, with recovery over 2–4 weeks; though in many cases patients remain asymptomatic [108–110]. Characteristic findings on OCT allow subclassification into three groups: (1) diffuse retinal thickening, (2) cystoid macular oedema and (3) foveolitis (Fig. 3), the latter including an orange-yellow central foveal lesion and associated with the longest persistence of scotomata [108]. Other findings include retinal haemorrhages, primarily venous vasculitic changes, and yellow sub-retinal spots [6]. Cases of rare neuro-ophthalmic complications have included optic neuropathy, neuromyelitis optica and abducens palsy [6].

Diagnosis relies upon dengue symptomatology, and confirmation by laboratory testing. In the first 5 days, real-time RT-PCR or NS1 antigen detection allow confirmation. More commonly confirmation occurs after 5 days, with ELISA-based detection of anti-Dengue IgM or seroconversion in paired acute and convalescent serum samples [97]. Workup of posterior segment pathology to identify and grade severity of complications as well as facilitate mechanistic understanding involves visual field testing and multimodal imaging [6]. The latter includes fundoscopy, fluorescein and indocyanine green angiography, OCT for subclassification, and more recently OCT angiography [108, 111]. Ocular manifestations resolve spontaneously in the majority of cases and evidence for topical or systemic treatment with corticosteroids lacks consensus [6]. Vaccines in the latter development stages are showing some promise [112].

### Zika* (Flaviviridae)

Zika virus (ZIKV) is an enveloped, positive-sense, single-stranded, RNA virus with an icosahedral capsid, and is a member of the Flaviviridae family [113, 114]. First discovered in Uganda, ZIKV has since disseminated widely throughout Africa, Asia, Pacific islands, South and Central America, causing notable outbreaks in Micronesia (2007), French Polynesia (2013) and the Americas (2015–16) [67, 109, 115].

Zika is principally transmitted via the bite of an infected Aedes mosquito (such as A. aegypti and A. albopticus), usually during daytime [116]. Human-to-human transmission can then occur via sexual contact, blood transfusions, organ donation and vertically from mother to foetus [117]. Findings of viral RNA in tears and conjunctival swabs from confirmed human cases highlight a potential ophthalmic route of transmission [118, 119].

Infected patients are typically asymptomatic, however up to 20% may develop a mild, self-limiting symptom triad of generalized maculopapular rash, arthritis or arthralgia and non-purulent conjunctivitis following a 3–14 day incubation period [117, 120, 121]. Though rare, severe neurological sequelae such as Guillain–Barré syndrome, myelitis and meningoencephalitis may develop ~5 days after acute disease onset [122, 123]. Infection anytime during pregnancy poses up to a 10% risk of the manifestation of congenital zika syndrome (CZS) due to vertical transmission, characterised by severe microcephaly, thin cerebral cortices, retinal disease, congenital contractures and early hypertonia and extrapyramidal involvement [124–126]. The most severe CZS phenotype occurs following first trimester exposure [120, 122, 127, 128].

Ocular involvement related to Zika virus may be acquired in the acute phase or present congenitally. In the acute phase, non-purulent conjunctivitis and retro-orbital pain are the most frequent ocular signs, both reported in 40% in a Brazilian case series of 57 ZIKV positive patients [129]. Reports of uveitis are uncommon (limited to under 20 published cases) and tend to have favourable visual outcomes without recurrence [130]. Anterior uveitis may be bilateral and non-granulomatous, and is associated with high intraocular pressures, corneal oedema and minimal fine keratic precipitates, responding effectively to topical corticosteroids and anti‐hypertensive drugs [131–133]. Posterior uveitis may manifest as a bilateral neuroretinitis, chorioretinitis with placoid or multifocal non-necrotizing lesions [134–136]. Reduced visual acuity can result from a unilateral acute idiopathic maculopathy occurring in the absence of uveitis, consisting of a unilateral perifoveolar greyish discoloration, and resolving within 6 weeks without treatment [137, 138].

Ocular manifestations of Zika have been described in up to 55% of infants with CZS [139, 140]. Two characteristic manifestations include a well-circumscribed chorioretinal atrophy with a hyperpigmented border and focal pigment mottling at the macula, occurring in up to 70% of children with ocular findings [116, 140, 141]. This posterior eye involvement is speculated to arise from viral breakdown of the blood–retinal barrier or axonal transport along nerves [141–144]. Numerous other ocular manifestations in CZS are possible, including various optic disc changes, retinal vascular disease, congenital glaucoma and cataract, lens subluxation, microphthalmia and iris coloboma, leading to multiple deficits in visual function (e.g. contrast, visual fields, accommodation and refractive errors) [140, 141, 145]. In addition, neuro-ophthalmic manifestations such as strabismus and nystagmus have been described [141]. Risk factors predicting ocular manifestations of CZS include maternal first trimester infection and presence of microcephaly at birth.

Confirmative diagnosis of ZIKV infection requires laboratory testing of whole blood or urine, and is also possible in other bodily fluids [117]. For instance, ocular fluid testing has aided diagnosis in ZIKV anterior uveitis [131]. Testing modalities include RT-PCR, ELISA and Zika plaque reduction neutralization test (PRNT) [146]. No specific vaccines or antiviral drugs are currently available, and treatment is supportive. Regarding CZS, the French Ministry of Health and CDC recommend mandatory comprehensive ophthalmic screening in all children born to a mother infected during pregnancy, prior to 1 month of age, with a further fundus exam repeated at 1 year [147, 148]. Early detection and expedient refractive correction can lead to immediate improvements in visual function in these children [149].

### West Nile (Flaviviridae)

West Nile virus (WNV) is an enveloped, positive-sense, single-stranded RNA virus of the Flaviviridae family, and a member of the Japanese encephalitis serocomplex of viruses [150]. WNV has five distinct phylogenetic lineages with only lineage one, distributed worldwide, and two, enzootic in Africa, known to cause disease in humans [150, 151]. The natural reservoir of WNV is birds, with transmission to humans occurring via Culex mosquitoes. Bird–mosquito–human transmission leads to the vast majority of disease in humans [150, 152].

Of patients who contract the virus, ~75% are asymptomatic, 25% develop West Nile fever (WNF) and <1% a neuroinvasive disease fatal in 10% [150, 153–155]. After an incubation period of around 2–14 days, WNF presents as a self-limiting, non-specific viral illness including fever, headache, fatigue, nausea and vomiting, lymphadenopathy and skin rash, that typically lasts less than a week [150]. Neuroinvasive disease causes meningitis, encephalitis, acute flaccid paralysis, movement disorders or other neurological manifestations [150, 156]. Advanced age is the strongest predictor of severe neurological manifestations and fatality [150].

**Fig. 4:**
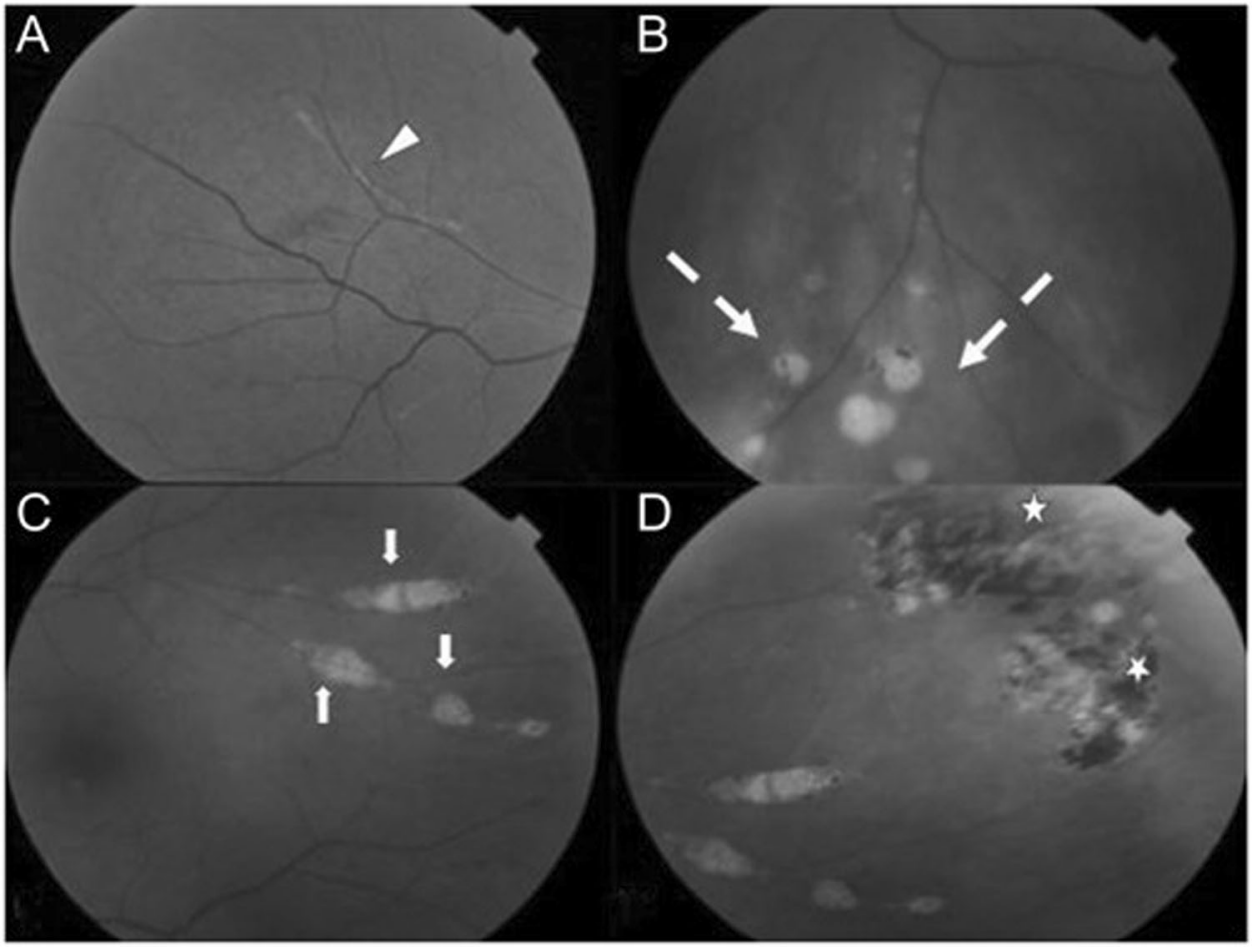
West Nile virus retinopathy. **A** Inflammatory vascular sheathing, **B** retinal necrosis, **C** multifocal chorioretinitis, **D** pigmented chorioretinal scars. Source: Hasburn [232] (CC-BY licence).

Multifocal chorioretinitis (Fig. 4D) is the most common ocular finding in WNV, typically bilateral, and occurring in 23 of 29 (79%) consecutive patients with laboratory confirmed WNV in the largest relevant prospective study to date [157]. Patients are frequently asymptomatic, though symptoms can include floaters, reduced visual acuity, redness, ocular pain, visual field defects or diplopia [157, 158]. The multifocal chorioretinitis involves 10–50 deep, flat, white or yellowish lesions per eye ranging from 100–1500 µm in diameter, typically arranged in a curvilinear cluster in the mid-periphery [109, 157]. Lesions tend to self-resolve without residual pigmentation and visual acuity typically returns to baseline [151, 158–160]. On fluorescein angiography, the active lesions are hypofluorescent initially and later hyperfluorescent. Inactive lesions have a target-like, rosette appearance [109, 161]. Chorioretinitis appears more frequently in those aged over 50 and those with diabetes mellitus [162, 163]. Other ocular manifestations include occlusive retinal vasculitis and intraretinal haemorrhages, optic neuritis, congenital chorioretinal scarring and uveitis (Fig. 4) [157, 158, 162].

Diagnosis is typically confirmed by laboratory testing using ELISA to detect IgM in serum of cerebrospinal fluid, and in limited scenarios supported by RT-PCR. There is no vaccine, treatment is supportive, with vector control as the mainstay of prevention [150].

### Yellow fever virus (Flaviviridae)

The yellow fever virus (YFV) is an enveloped, positive-sense, single-stranded RNA virus of the Flaviviridae family. It is an arbovirus transmitted to humans primarily through the bite of infected Aedes or Haemagogus species mosquitoes, and principally maintained by a sylvatic (jungle) transmission cycle between mosquitoes and non-human primates and mosquito–human transmission cycles in urban areas, the latter leading to outbreaks of yellow fever (YF) in human populations. Dozens of countries in both Africa and South and Central America are endemic for YF, leading to an estimated 30,000–60,000 fatalities per year [164].

The majority infected with YFV are asymptomatic or exhibit an initial phase of non-specific viral symptoms after 3–6 days (fever, myalgia, nausea and vomiting), which then resolves within days. After less than a day of resolution, 15–25% of patients develop a toxic form of the disease, with fever, a haemorrhagic diathesis and multi-organ failure (especially renal and hepatic) leading to a 50% mortality rate [165, 166].

A common ocular finding in the initial phase is conjunctivitis, whilst scleral icterus is often a feature of the toxic phase [109, 166]. A small number of case reports suggest other ocular manifestations, with two intensive care patients reported in Brazil noted to present with bilateral increased choroidal thickness. One subsequently develops bilateral retinal vein congestion. The other developed bilateral mid-peripheral 360° detachment of the choroid and yellowish sub-retinal lesions. In another reported case, unilateral retinal oedema, macular exudates and haemorrhages were found during the convalescent stage, potentially due to immune-mediated causes [167].

There also exist case reports of ocular disorders associated with YFV vaccination, especially when the live YFV vaccine is combined with other vaccines, such as *Neisseria* Meningitidis, Hepatitis A/B or Typhoid. The reported complications are varied and include uveitis, unilateral optic neuropathy, evanescent white dot syndrome, multifocal choroiditis, arteriolar occlusion and even a Vogt–Koyanagi–Harada-like disease (with accompanying serous retinal detachment and choroidal thickening) [109, 168, 169]. These conditions are rarely reported and most often self-limiting with a favourable course over a few weeks with simple supportive anti-inflammatory treatment.

Diagnosis of YFV relies on PCR of blood or urine samples in the early stages, and ELISA or PRNT to detect antibodies in later stages. Treatment is mainly supportive, with a strong emphasis on prevention through public health measures and vaccination [166, 170].

### Chikungunya (Togaviridae)

Chikungunya virus (CHIKV) is an enveloped, positive-sense, single-stranded RNA virus belonging to the genus Alphavirus of the family Togaviridae. CHIKV has been identified in over 60 countries in Asia, Africa, Europe and the Americas, and several epidemics have been reported in the past 20 years commonly during monsoon season. CHIKV is principally transmitted by the bite of the mosquitoes of the *Aedes* genus—*Aedes aegypti* and *Aedes albopictus* are the primary vectors [171]. Transplacental transmission has been reported, without significant outcomes for mother and child [172].

CHIKV infection results primarily in an acute fever with a severe polyarthralgia predominating in the distal extremities that may persist for several weeks or months [173]. Cervical or generalized lymphadenopathy may be present, along with various mucocutaneous manifestations. Neurologic complications such as meningoencephalitis or other multi-organ failure are rare, though described in the immunocompromised and at the extremes of age. There is an overall mortality rate of 1 in 1000, largely restricted to the elderly [174]. However, often symptoms are mild and may resolve unrecognized, or be misdiagnosed in areas where dengue occurs.

**Fig. 5:**
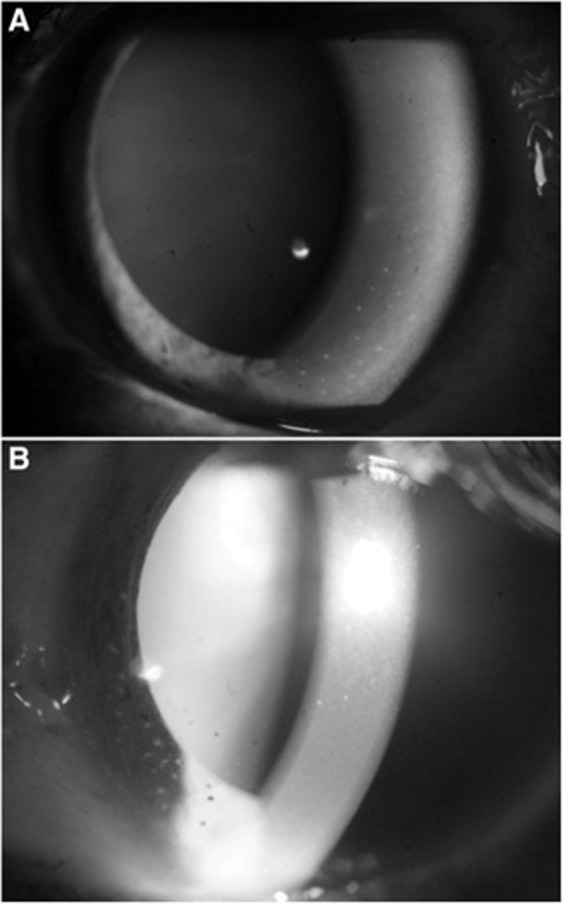
Chikungunya, slit lamp anterior segment photos with 1+ cells and 2+ flare evident in the anterior chamber of both eyes. **A** Pigmented keratic precipitates in the inferior cornea, **B** stellate keratic precipitates. Source: Mahendradas [233] (CC-BY Licence).

CHIKV has a variety of ocular manifestations ranging from conjunctivitis to retinitis. Photophobia, conjunctival hyperaemia and retro-orbital pain are frequent in the acute phase, and may be isolated without other ocular involvement. Anterior uveitis is the most frequently reported ocular complication (one in three ocular cases), appearing 4–12 weeks after disease onset [175]. The anterior uveitis may be either granulomatous or non-granulomatous, and may be associated with elevated intraocular pressure, or more infrequently with posterior synechiae. A Fuchs’ uveitis phenotype of fine stellate keratic precipitates is visualised on confocal microscopy, with a diffuse distribution over the posterior cornea (Fig. 5) [109]. Although presence of CHIKV RNA has been documented by PCR analysis of aqueous samples in some patients with anterior uveitis and previous CHIKV fever, it is unclear whether this is pathogenic or merely a remnant of recent systemic illness [176]. The uveitis responds well to steroid drops and anti-glaucoma drops if required, and complete resolution is achieved in <3 weeks [177].

**Fig. 6:**
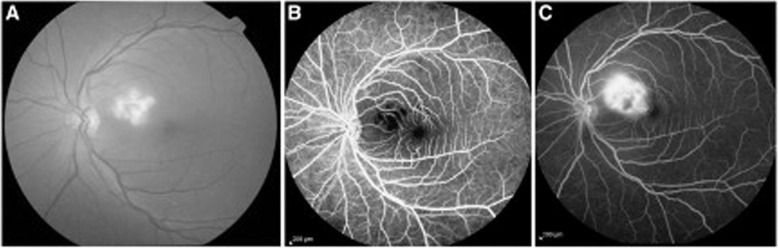
Chikungunya, retinal manifestations. **A** Fundus image of the left eye showing confluent area of retinal whitening suggestive of retinitis. **B**, **C** Fundus fluorescein angiography showing the posterior pole in early and late hypofluorescence, respectively. Source: Mahendradas [233] (CC-BY Licence).

Posterior uveitis often presents weeks after the acute symptoms, as retinitis, multifocal choroiditis or neuroretinitis. Neuroretinitis involves exudative haemorrhagic lesions essentially localized to the posterior pole, which can be associated with intra-/sub-retinal macular oedema [178]. The lesions are hypofluorescent in the early phases and hyperfluorescent in the late phases of fluorescein angiography (Fig. 6). The optic disc is hyperaemic, and the vitreous shows a slight inflammatory reaction. Empiric treatment with acyclovir (ineffective against RNA viruses), systemic steroids and non-steroidal anti-inflammatory drops can lead to favourable outcomes in <2 months, though recurrence of retinitis has been reported [177, 179, 180].

Optic neuropathy is evident in 10% of cases with ocular involvement, developing typically 1 month after disease onset [109]. In one case series of 14 CHIKV+ patients with clinical features suggestive of optic neuritis, all complained of a severe decrease in visual acuity and earlier initiation of treatment with IV methylprednisolone was associated with improved visual recovery [181]. The underlying mechanism may involve direct viral involvement (as in some cases, the onset of optic neuropathy is coincident with that of systemic signs) or an immune mechanism (as in the majority, where delayed onset, bilateral involvement and a good response to steroid treatment has been observed). Other ocular lesions have also been described: keratitis, scleritis and episcleritis, lagophthalmos and oculomotor palsies [109]. The presence of CHIKV has been demonstrated in the cornea during the viraemic phase, resulting in exclusion from corneal donation in endemic areas [182].

Formal diagnosis of CHIKV in the initial 8 days of acute infection is done by RT-PCR or direct isolation of virus/viral antigens, following which serological testing with ELISA is recommended [183]. Case reports highlight that RT-PCR may detect CHIKV RNA in aqueous fluid [184]. Antiviral drug and vaccine development are underway [185, 186].

### Crimean Congo* (Nairoviridae)

Crimean-Congo haemorrhagic fever virus (CCHFV) is an enveloped, circular, negative-sense, single-stranded RNA virus in the Nairoviridae family, which causes an acute viral haemorrhagic fever. Transmission of the virus is primarily through tick bites belonging to the genus Hyalomma, which are widely distributed in Asia, Africa and Eastern Europe [187]. Transmission is also reported to occur by contact with patients or animals with a high viral load, thus posing a high risk of transmission in healthcare environments [188].

An estimated 90% of patients are oligo- or asymptomatic whilst the remaining 10% can present with the more severe form of disease described in many studies [189]. The disease manifests as sudden onset fever, myalgia and dizziness. Some develop gastro-intestinal symptoms such as vomiting and stomach pain. After 3–5 days, the haemorrhagic phase starts including bleeding (severe bruising and epistaxis), confusion and hepatomegaly. Fatality rates for hospitalised patients are documented as ranging from 9 to 50% [190].

Only one study has formally characterised the ocular findings in CCHFV patients after previous studies reported conjunctival injection and photophobia [191]. In a case series of 19 patients, none had ocular complaints, but examination showed that 14 (73.7%) had ocular abnormalities. Subconjunctival haemorrhage was present in 12 (63.2%) patients and retinal haemorrhage was present in 7 (36.8%) patients. Subconjunctival haemorrhage was petechial, occurring most often at the nasal quadrant and peri-limbal area of both eyes. Retinal haemorrhage was dot-like or triangular at the paramacular and peripapillary area. The ocular findings did not appear to correlate with disease severity. Both types of haemorrhage had resolved completely at 1 month follow-up. The results of this small study indicate that CCHF should be considered in the differential when subconjunctival or retinal haemorrhages are seen along with fever in travellers from endemic areas [191].

Diagnosis is via serum RT-PCR in the early stages of disease or ELISA late in disease and treatment is largely supportive with some evidence that ribavirin shows some benefit [192, 193]. After people of working age exposed to tick populations, healthcare workers are the second most-affected group [192]. Knowledge of CCHFV may therefore help prevent nosocomial spread of the virus.

## Droplet

### Coronavirus disease 2019 (COVID-19)* (Coronaviridae)

Severe acute respiratory syndrome coronavirus 2 (SARS-CoV-2) is an enveloped, single-stranded, positive-sense, RNA virus of the family coronaviridae [194]. SARS-CoV-2 emerged as the cause of coronavirus disease 2019 (COVID-19) in December 2019 in Wuhan, China. The subsequent pandemic of this highly transmissible virus has caused in excess of 1250,000 deaths at the time of writing, and poses an ongoing threat to global public health [195]. The main modes of transmission are respiratory droplets and direct contact [196].

COVID-19 has a viral pneumonia picture. Early symptoms occur after a median incubation period of 4 days, most commonly fever, cough and fatigue [197]. In a proportion of cases, a severe form of the disease ensues, with progression to an acute respiratory distress syndrome picture and type 1 respiratory failure. In such cases, hospitalisation is required for oxygen supplementation, and/or intubation and ventilation. Increased age and co-existing illness are associated with increased severity of disease [197].

Viral RNA has been detected in ocular secretions [7, 198]. Several reports have suggested that SARS-CoV-2 can cause a mild follicular conjunctivitis, with symptoms including conjunctival hyperaemia, chemosis, epiphora and increased secretion. Twenty-three cases have been described so far in published literature, and three cases in pre-published studies [7, 197–201]. The largest study reporting cases described conjunctival congestion in 9 of 1099 patients (0.8%) [197]. There has been only one study showing retinal changes in COVID-19 patients [202]. Twelve COVID-19 patients examined in the study all displayed hyper-reflective changes in retinal ganglion cell and inner plexiform layers binocularly using OCT. Four patients had cotton wool spots and microhaemorrhages but no signs of intraocular inflammation. Despite OCT changes in the retina, no visual acuity nor pupillary reflex changes was found.

RT-PCR of oropharyngeal and nasopharyngeal swabs is the mainstay of testing, with other sources sampled less commonly. However, false negatives present a problem and so clinical suspicion based on symptomatology, biochemistry and imaging findings play an important role. Many clinical trials are already underway to assess potential treatment options and trials have begun for candidate vaccines [202].

### Influenza A H1N1 (Orthomyxoviridae)

Influenza A H1N1 is an enveloped, negative-sense, single-stranded RNA virus of the orthomyxoviridae family. The subtype of Influenza A relates to cell surface glycoproteins. Influenza A undergoes continuous antigenic drift causing seasonal influenza, and sporadic antigenic shifts causing pandemics [203]. Notable pandemics include the 1918 Spanish flu and 2009 Swine flu (A(H1N1)pdm09 virus). Since then H1N1 has been identified in normal seasonal flu along with H3N2 [204]. A global average of 294,000–518,000 respiratory deaths is associated with seasonal influenza annually [205]. The virus spreads primarily via droplet transmission though there is a role for indirect contact transmission [206]. Ex vivo cultures have also found that the A(H1N1)pdm09 subtype has the ability to replicate in the human conjunctiva highlighting the conjunctiva as a potential route of infection [207].

Common symptoms include fever, cough, sore throat, coryza, myalgia, headaches and fatigue, and the disease is usually self-limiting [208]. The most common complications are viral pneumonia or secondary bacterial pneumonia. Other complications include myositis, rhabdomyolysis, myocarditis, pericarditis, encephalitis, transverse myelitis and Guillain-Barre syndrome [208]. In elderly and other high-risk individuals, deterioration of underlying cardiovascular, pulmonary and renal function may lead to irreversible changes and death [209].

Ocular involvement in influenza A virus disease is common often involving mild conjunctivitis to reports of more severe eye involvement. True prevalence of ocular manifestations is difficult to ascertain from isolated studies due to lack of thorough ophthalmoscopic examinations during influenza outbreaks, and the benign nature of the disease in most patients. This accounts for the paucity of literature and detailed retinal findings [209].

**Fig. 7:**
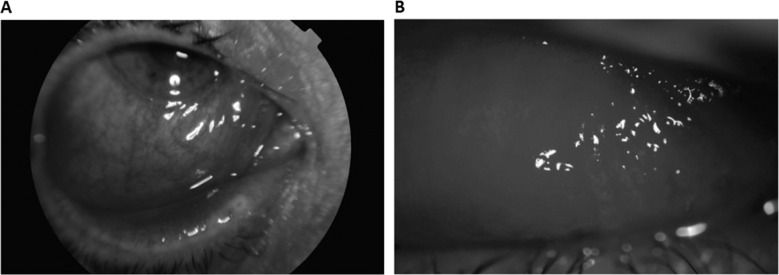
Influenza virus H1N1, severe ocular cases. **A** Lower chemosis and severe conjunctival hyperaemia associated with significant eyelid oedema, **B** petechiae and significant upper sub-tarsal follicles. Source: Lopez-Pratz [234] (CC-BY Licence).

A growing body of evidence highlights the potential ocular findings in influenza A H1N1 disease. A case series of 89 patients with H1N1 infection found that acute conjunctivitis was the most common finding occurring bilaterally in 65.17% of patients and unilaterally in 12.36% patients [210]. Features of significant eyelid oedema, conjunctival hyperaemia, watery discharge and moderate chemosis with sub-tarsal follicles were evident in most patients. Ten cases of conjunctivitis were severe and two were haemorrhagic with sub-tarsal petechial haemorrhage, with such findings imaged elsewhere (Fig. 7). The condition resolved with topical NSAIDs and topical ganciclovir. Corneal involvement was present in eighteen (20.22%) patients. Fluorescein staining revealed multiple bilateral corneal erosions that resolved by day 7. Iridocyclitis is a much rarer reported anterior segment finding [209].

Involvement of the posterior segment is less frequent. However, there have been reports of uveal effusion syndrome, retinopathy, retinitis and optic neuritis [210–216]. Uveal effusion syndrome occurs around 2 days after flu-like symptoms. Painful red eye, reduced visual acuity, a quiet shallow anterior chamber, and swelling of the posterior chamber and vitreous cells indicative of sub-retinal exudation, are reported [210, 211]. Treatment with topical and systemic corticosteroids improved the condition in 10 days [210]. Visual defects from retinopathy usually self-resolve in 3 weeks; however, steroids may play a role in the treatment of severe cases [210, 212, 214]. One case of retinal vasculitis occurred without recovery of vision despite steroid therapy [217]. Optic neuritis has been reported with steroid therapy improving the condition [210, 216, 218]. Reports of encephalopathy associated with cortical visual loss and oculomotor palsy is also possible [219–221].

Diagnosis is made clinically except in certain scenarios where testing may influence clinical decisions, such as whether to initiate antiviral treatment, perform other diagnostic testing or to implement infection prevention and control measures [222]. Recommended diagnostic tests include rapid molecular assays for outpatients and RT-PCR is recommended for hospitalised patients, using nasopharyngeal, nasal or throat swabs [223]. Awareness of ocular symptoms in influenza A virus infection may facilitate early intervention with steroids, antiviral drugs and topical treatments to prevent long-term visual damage in severe cases of H1N1. Both strains of seasonal influenza H1N1 and H3N2 are targeted in the annual influenza vaccinations [200, 201].

## Prevention and treatment

These emerging viruses pose a major public health risk due to their known pandemic potential and/or insufficient counter measures. Notably, these pathogens are all RNA viruses, whose error prone RNA-dependent RNA polymerase enzymes result in inherently high mutability and capacity for host immune evasion, which may underlie their pandemic potential [224]. Continued discovery and development of new antiviral medications and vaccines are a research priority for global health, particularly as new pathogens periodically emerge and old ones evolve to evade current therapeutic agents. For the viruses discussed in this review, the availability of approved antiviral therapies and vaccines is limited (with approval only for influenza A H1N1 and Ebola vaccines); however, a number is currently under development.

In the absence of specific antiviral drugs or immunisation, precautionary strategies are the mainstay for curbing transmission. These include vector control and exposure limitation, screening and prophylaxis of exposed individuals, isolation of infected individuals and potential contacts, precautions depending on transmission route, and both personal and environmental hygiene and decontamination. Strategies for pandemic preparedness also need to be capable of mitigating risks posed by yet to emerge, unknown viruses, as reflected by inclusion of “Disease X” on WHO’s shortlist of blueprint priority diseases.

Many of these viruses confer potential risk to eye care providers. Isolation of viral RNA from ocular secretions has been confirmed in humans for Ebola, Marburg, Zika, Chikungunya, COVID and influenza A H1N1, as well as in animal models of LF [7, 20, 21, 30, 118, 119, 184, 198, 207, 225, 226]. This possibility remains to be further investigated for Nipah, RVF, Dengue, West Nile and Crimean Congo virus. Together, these highlight the need for extra precautions during ophthalmic procedures in certain cases, along with advisory eye protection to limit contagion. Eye specialists are at particular risk due to the close proximity during eye examination and the potential for ‘air puff’ tonometry to generate droplets or aerosols [227]. General precautions in ophthalmic practice includes installation of transparent shields on slit-lamps, disinfection of surfaces and instruments after each use, donning of protective equipment, appropriate triaging and implementation of telemedicine where appropriate [228, 229]. Given the variations in practice patterns, practitioners should heed their respective best-practice guidelines for infection control.

## Summary

The threat of emerging viral disease has been made all the more clear in the midst of the current COVID-19 pandemic. Further, the possibility that a yet unknown pathogen could cause a future epidemic has also been acknowledged by the WHO on their shortlist of blueprint priority diseases as ‘Disease X’. It has been estimated that there are 1.67 million unknown viruses circulating in animal reservoirs and there remains the potential for them to evolve and transmit to humans [230]. It is interesting that the majority of new ‘threats’ have ocular manifestations.

Ophthalmologists should therefore be mindful of the variation of presentations of eye diseases in this context. The COVID-19 pandemic and studies of other viral diseases highlight that globalisation and modernisation play a significant role in the spread of viral disease and consequently returning travellers could present with manifestations of both current known and novel viral diseases. An awareness of the ocular manifestations of emerging viral diseases can therefore enable eye specialists to facilitate swift diagnosis, improve clinical outcomes and contribute to wider public health efforts during outbreaks.
